# Supplementary material for: Delayed development of basal spikelets in wheat explains their increased floret abortion and rudimentary nature
Source: J Exp Bot. 2023 Jun 20;74(17):5088–103. doi: 10.1093/jxb/erad233 (PMC10498016; doi:10.1093/jxb/erad233)
Supplement: erad233_suppl_Supplementary_Figures_S1-S3 [file erad233_suppl_supplementary_figures_s1-s3.pdf]

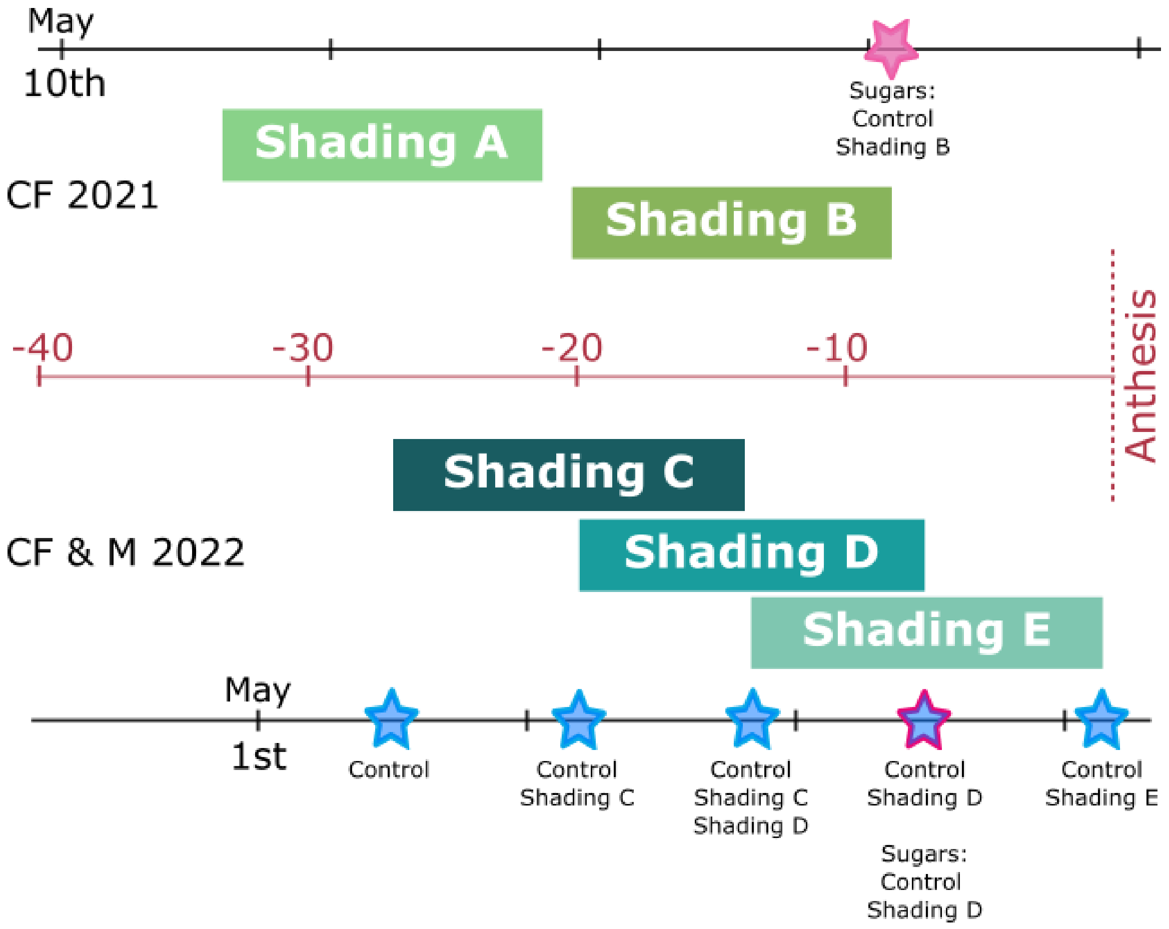

Fig. S1. Schematic of floret development and sugar sampling in 2021 and 2022. Blue stars indicate floret collection dates, pink star or outline indicate sugar collection dates.

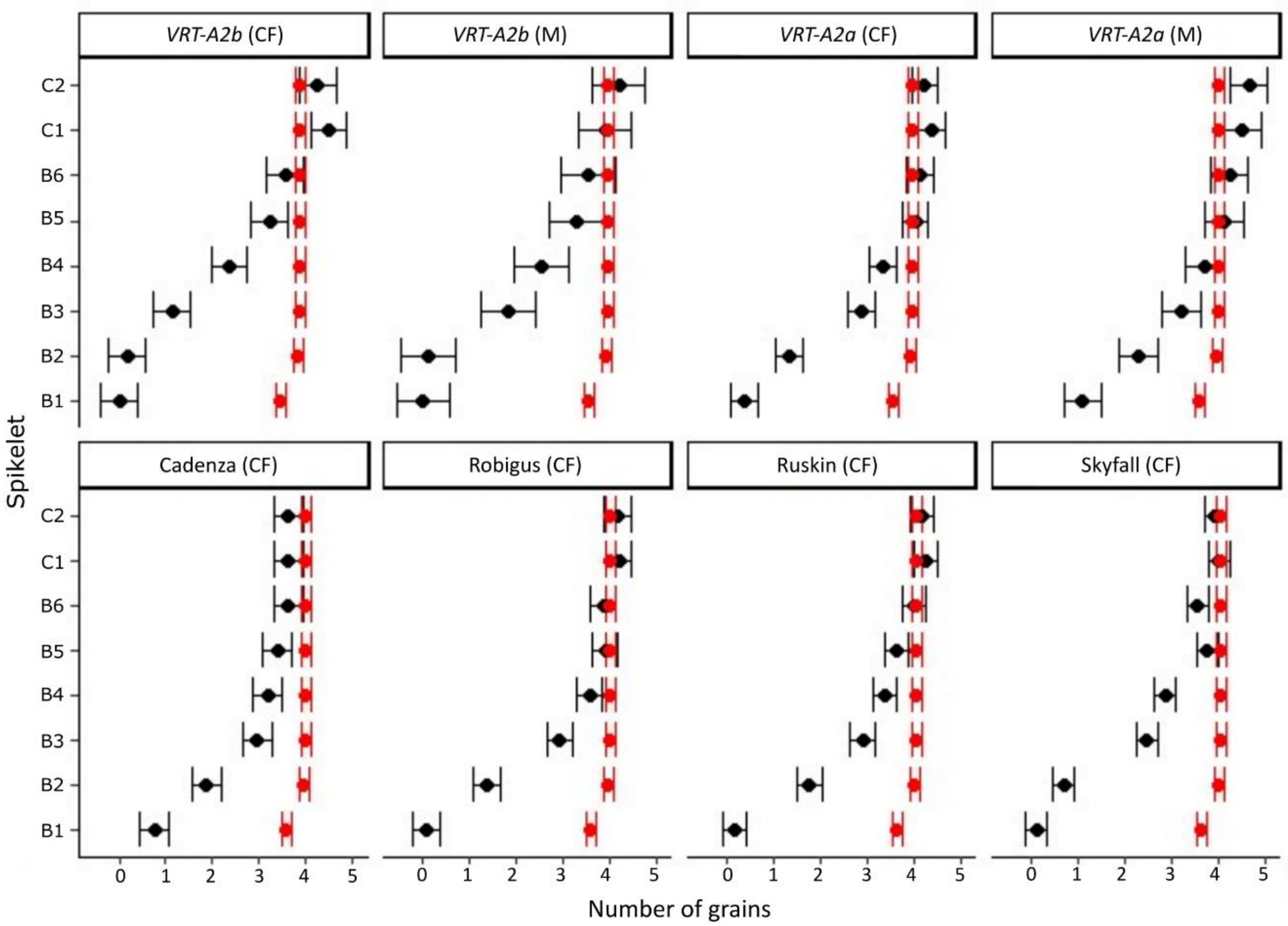

Fig. S2. Predictions of grains per spikelet using floret count per spikelet pre-abortion (20 DPA) and deducting same number of florets per spikelet (4 florets) across all spike positions. Black = Number of grains per spikelet recorded in mature spike, Red= predicted grains/spikelet. B = Basal (spikelet), C = Central (Spikelet).

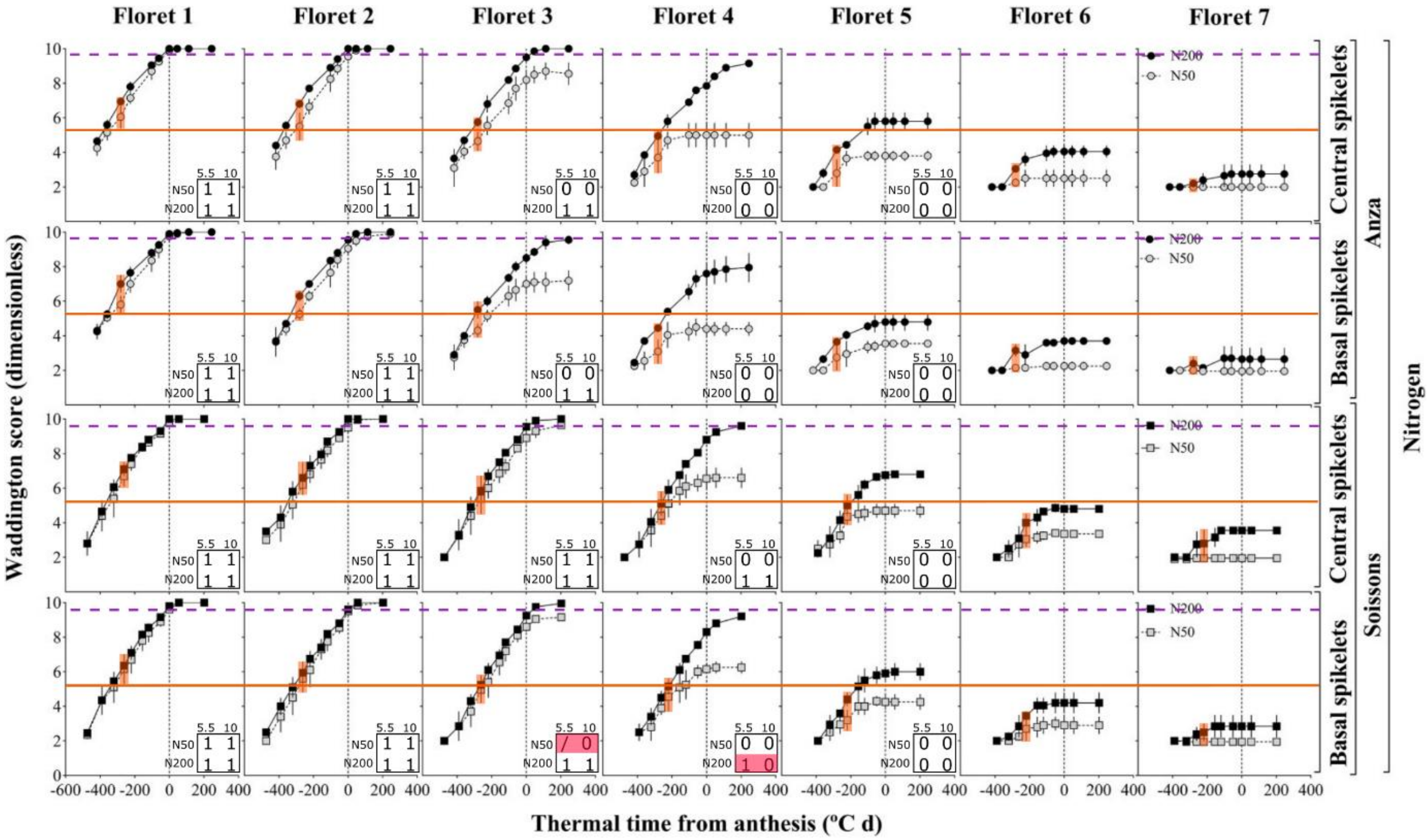

Fig. S3. Re-analysis of Ferrante et al (2020) Figure 9. Orange bar was added to mark Waddington stage 5.5 and the data points corresponding to maximum floret number stage pre-abortion (circa -270 ˚C d) were highlighted in orange to determine if florets had reached Waddington stage 5.5 at that time. Purple dotted line indicates cut-off for florets considered to have reached maturity (W10). Out of the 56 floret development traces, 22 reached Waddington stage 10 by the end of the time course and all of these had passed Waddington stage 5.5 at -270 ˚C d (which corresponds to maximum floret number stage according to Figure 8 (Ferrante et al., 2020)). Of the 34 florets that did not reach Waddington stage 10 at maturity, 91% had not reached W5.5 at -270 C˚. Only floret 4 of the basal spikelet in Soisson (high nitrogen) had reached W5.5 but then did not develop until the W10 cut-off (purple line) while the development stage of floret 3 in the same genotype and spikelet at nitrogen level 50 could not be accurately scored. Decisions on floret development are indicated in box on bottom right of each panel. If florets reached the stage they were scored 1, if they didn't they were scored 0. Red shading indicates that floret development at W5.5 does not match their development stage at W10. As all florets were infertile beyond floret 5 we stopped analysis beyond floret 5. Original legend from Ferrante et al (2020), "*Dynamics of the floret development from floret 1 (F1, floret primordium closest to the rachis) to floret 7 (F7, floret primordium most distal to the rachis) in each of the two spikelet categories considered of the main-shoot through thermal time from anthesis (negative values represent the period before anthesis) in the N experiments for Anza and Soissons. Grey and black symbols correspond to N50 (50 KgN ha^{-1}) and N200 (200 KgN ha^{-1}). Each data-point is the average of all replicates across two growing seasons and within each replicate the value was the average of 10 (2010-11) and 5 plants (2011-12), bars represent the standard error of the means (not visible in some cases as it was smaller than the body of the symbol).*"
